# Lactic Acid Bacteria as Antimicrobial Agents: Food Safety and Microbial Food Spoilage Prevention

**DOI:** 10.3390/foods10123131

**Published:** 2021-12-17

**Authors:** Salam A. Ibrahim, Raphael D. Ayivi, Tahl Zimmerman, Shahida Anusha Siddiqui, Ammar B. Altemimi, Hafize Fidan, Tuba Esatbeyoglu, Reza Vaseghi Bakhshayesh

**Affiliations:** 1 Food and Nutritional Sciences Program, North Carolina A&T State University, Greensboro, NC 27411, USA; rdayivi@aggies.ncat.edu (R.D.A.); tzimmerman@ncat.edu (T.Z.); 2 Department of Biotechnology and Sustainability, Technical University of Munich (TUM), 94315 Straubing, Germany; s.siddiqui@dil-ev.de; 3 DIL e.V.—German Institute of Food Technologies, 49610 D-Quakenbrück, Germany; 4 Department of Food Science, College of Agriculture, University of Basrah, Basrah 61004, Iraq; ammaragr@siu.edu; 5 Department of Nutrition and Tourism, University of Food Technologies, 26 Maritza Blvd., 40002 Plovdiv, Bulgaria; hfidan@abv.bg; 6 Institute of Food Science and Human Nutrition, Gottfried Wilhelm Leibniz University Hannover, Am Kleinen Felde 30, 30167 Hannover, Germany; esatbeyoglu@lw.uni-hannover.de; 7 Department of Food Biotechnology, Branch for Northwest & West Region, Agricultural Biotechnology Research Institute of Iran, Agricultural Research, Education and Extension Organization (AREEO), Tabriz 5355179854, Iran; reza.vaseghi68@gmail.com; 8 Department of Food Science and Technology, University of Tabriz, Tabriz 5166616471, Iran

**Keywords:** antimicrobial, lactic acid bacteria (LAB), bacteriocin, biopreservation, foodborne pathogens

## Abstract

In the wake of continual foodborne disease outbreaks in recent years, it is critical to focus on strategies that protect public health and reduce the incidence of foodborne pathogens and spoilage microorganisms. Currently, there are limitations associated with conventional microbial control methods, such as the use of chemical preservatives and heat treatments. For example, such conventional treatments adversely impact the sensorial properties of food, resulting in undesirable organoleptic characteristics. Moreover, the growing consumer advocacy for safe and healthy food products, and the resultant paradigm shift toward clean labels, have caused an increased interest in natural and effective antimicrobial alternatives. For instance, natural antimicrobial elements synthesized by lactic acid bacteria (LAB) are generally inhibitory to pathogens and significantly impede the action of food spoilage organisms. Bacteriocins and other LAB metabolites have been commercially exploited for their antimicrobial properties and used in many applications in the dairy industry to prevent the growth of undesirable microorganisms. In this review, we summarized the natural antimicrobial compounds produced by LAB, with a specific focus on the mechanisms of action and applications for microbial food spoilage prevention and disease control. In addition, we provide support in the review for our recommendation for the application of LAB as a potential alternative antimicrobial strategy for addressing the challenges posed by antibiotic resistance among pathogens.

## 1. Introduction

Foodborne diseases and food spoilage organisms continue to exert negative impacts on public health and the food industry. Foodborne disease outbreaks have resulted in a high rate of mortality, along with the high financial burden stemming from healthcare costs. Moreover, the soaring numbers of confirmed cases of foodborne illnesses are very alarming, despite the availability of the hazard analysis and critical control point (HACCP) system. The Council for Agricultural Science and Technology reported that 6.5–33 million cases of human ailments were associated with food, with a reported fatality rate of 9000 cases annually in the U.S. (Food and Agriculture Organization) [1]. In the European Union, the most common causes of foodborne infections are associated with the following bacteria: *Campylobacter jejuni*, *Listeria monocytogenes*, *Salmonella enterica*, *Escherichia coli*, *Listeria monocytogenes*, and *Staphylococcus aureus*, as well as viral pathogens, such as noroviruses and rotaviruses [2]. Interestingly, most food groups, such as eggs, meat, dairy products, fruits, vegetables, seafood, and poultry, cause outbreaks of foodborne diseases. According to the World Health Organization (WHO), if drastic measures are not taken by 2050, the global death rate from foodborne illnesses will increase to an estimated 10 million people annually. Such global, prevailing foodborne infection rates thus warrant a systematic approach for the elimination, prevention, and reduction in pathogenic bacteria in foods via the application of novel antimicrobial agents [2].

Due to their high nutritional content, moisture, and neutral pH, animal-derived foods are highly perishable. The processing of food using appropriate methods is critical to preserving food quality and maintaining safety. Applicable preservation methods for foods include low-temperature preservation methods, such as refrigeration and freezing techniques, and high-temperature preservation methods, such as pasteurization, sterilization, and chemical preservation [3,4,5,6]. However, because chemical preservatives are either proscribed or not accepted by consumers, there has been an increase in usage of biological preservatives for the enhancement of food safety and food quality. For example, biopreservation using natural microflora, such as lactic acid bacteria (LAB), has been recommended in lieu of the conventional use of chemical preservatives. Microbiota are safer, promote nutritional enhancement, and are considered to be clean label additives [7]. Biopreservation has received special attention among alternative food storage technologies. Biopreservation promotes shelf-life extension and consequently improving hygienic consistency, without negatively impacting the organoleptic characteristics and nutritional properties of perishable foods [7].

Numerous fermented foods naturally contain lactic acid bacteria (LAB) and bacteriocins, with LAB acting as a natural, vital biopreservative agent. Moreover, some LAB have produce important metabolites, such as reuterin, bacteriocins, diacetyl, reutericyclin, organic acids, acetoin, and hydrogen peroxide, which are highly potent biopreservative agents [8]. Most LAB inhibit the growth of some foodborne pathogens and spoilage microorganisms. Bacteriocins constitute a diverse group of antimicrobial peptides that are ribosomally synthesised and can destroy closely related microbial strains. Bacteriocins to inhibit a variety of pathogenic bacteria in several food matrices, such as in vegetables, meat, and cheese [2,9]. Ye et al. (2021) [2] demonstrated that a novel bacteriocin produced by *Lacticaseibacillus paracasei* ZFM54 had a broad-spectrum inhibitory action against targeted foodborne pathogens, such as *Listeria monocytogenes*, *Micrococcus luteus*, and *Salmonella typhimurium,* through pore formation in the cell membrane. The 1–3.3 KDa bacteriocin produced by *Lacticaseibacillus rhamnosus* 1.0320 demonstrated antimicrobial action against Gram-negative and Gram-positive bacteria by several mechanisms. These antimicrobial mechanisms include an increase in cell membrane permeability, pore formation on the surface of cell membranes, a change in transmembrane pH gradient, dissipation of the cytoplasmic membrane potential, and the destruction of cell membrane integrity. The consequence of these processes is cell content loss and eventually cell death. However, the mechanisms of the actions of other bacteriocins are still unclear [2].

This review thus aims to enhance the current knowledge about the antibacterial activity of LAB strains and potential use of bacteriocins for future applications, primarily in the dairy industry. The methodology employed in this review included literature searches conducted in PubMed, Web of Science, Web of Knowledge, Scopus, and Google Scholar that were relevant to the subject of antimicrobial properties of LAB. No range of years was specified; however, only peer-reviewed papers were considered for inclusion in this review.

## 2. The Microbial Ecosystem

The microbial ecosystem is uniquely balanced with diverse microorganisms interacting with each other, which ultimately influences other microorganisms in the population. The ecological system of microbes includes several host–pathogen interactions, namely predation, commensalism, synergism, parasitism, inhibition, and competition with food [3]. In recent decades, the meat and dairy industries have employed LAB as starter cultures in many applications. In the food industry, strains of both homofermentative and heterofermentative bacteria are used with strictly defined properties and cultivation conditions for the production of yogurt, sour cream butter, various types of cheese, and fermented beverages. Mixed cultures in food typically comprise of a consortium of microorganisms that interact with one another, thereby enhancing their metabolic activity to produce desirable outcomes on product quality and safety. 

Microbial growth is also impacted by several environmental factors. Some microorganisms have symbiotic interactions with each other. For example, one type of microorganism could synthesize chemical compounds that serve as an important primary resource for another microorganism to metabolize. This relationship is generally termed as a symbiotic interaction. This growth is usually found in food matrices that contain two or more microbes. Another example of an interaction between microorganisms is observed when two or more microbial types have hindered growth as a result of their interaction [4]. Microorganisms do not exist as pure cultures. Their physiological structures are determine by a complex interaction of ecological and environmental factors that exist between members in a diverse taxonomical community. Mixed cultures are fundamentally used for studying microbial interactions. A classic example uses mixed microbial isolates for population dynamic studies, such as mutualism and competition. Recently, synthetically mixed cultures were verified for specific characteristics and functions, such as biofuel generation and bioremediation, and these are useful for industrial applications. Mixed culture studies, which offer much evidence-based information linked to cultivation of microbial mixtures, are an excellent focal point for studying various biochemical interactions and ecological transformations looking for, and globally cultivating, these untreated microorganisms [4,5]. Sieuwerts et al. (2018) [6] studied the presence of mutualistic symbiotic interactions between *Lactiplantibacillus plantarum*, *Lactobacillus sanfranciscensis*, and *Saccharomyces cerevisiae* in sourdough fermentation, hypothesizing that the consumption of lactic acid by *S. cerevisiae*, or the growth stimulatory action of yeast, resulted in de-acidification of the growth medium, thereby leading to the growth of *L. sanfranciscensis.* As a result of their analysis, it was evident during the pre-fermentation stages that the carbon dioxide produced by *S. cerevisiae* and the use of oxygen led to an increase in the activity of *L. sanfranciscensis* in MRS (De Man, Rogosa, and Sharpe) agar. Another important indicator was the stimulation of *L. sanfranciscensis*, that was also observed in the process of dough fermentation. The stimulation was not explained by the deacidification of the agar plate, so it must have been a result of the vitamins provided by *S. cerevisiae*. The co-culturing of *L. sanfranciscensis* and *S. cerevisiae* was shown to enhance fermentation due to the consumption of lactic acid by yeast in the dough environment, which ultimately stimulated the growth of LAB in the sourdough, thereby drastically slowing down the rate of acidification. On the other hand, *S. cerevisiae* could only stimulate *L. plantarum* only when specific carbon sources, such as lactose, glucose, and fructose, but not sucrose, galactose, starch, and maltose, were available [6].

## 3. LAB Affect the Growth of Microorganisms

The overall objective of shelf-life extension is to keep food products safe and stable, and this could generally be achieved by controlling the growth of spoilage microorganisms and pathogenic bacteria. In addressing and controlling pathogenic growth, an antimicrobial agent, such as nisin, or two or more antimicrobial agents could be employed synergistically against the target organism. This concerted action is effective, as an individual agent may fail to completely prevent the growth of the target microorganism. It is noteworthy that the action of these antimicrobial agents does not negatively impact the nutritional and sensorial qualities of foods, thereby preserving their physicochemical structure. LAB are beneficial when added to food because they are able to: (1) prevent the growth of harmful enteric pathogens, (2) supply enzymes, (3) eliminate toxic food elements in the intestine, (4) promote immunomodulatory action stimulate the immune system, and (5) enhance the peristaltic action of the gastrointestinal tract [5].

Antagonistic activity against intestinal and food pathogens is an essential part of the probiotic properties of LAB, and antimicrobial activity is a sought after quality in selecting strains. LAB possess antimicrobial properties that target fungi and several Gram-negative and Gram-positive bacteria [6], and are therefore important for the fermentation, preservation, and storage of food. The antimicrobial properties of LAB strains are mediated by the antimicrobial molecules produced by these strains. These antimicrobials can be divided into three primary groups: (a) peptidic or proteinacious bacteriocins; (b) organic acids (butyric acetic acids and lactic acid); (c) other small molecules, for example diacetyl, hydrogen peroxide, acetaldehyde, acetoine, reuterin, and reutericyclin [7]. These are elaborated as follows:

(a) Bacteriocins are antimicrobial peptides generated from various types of bacteria, including LAB [8]. Bacteriocins are generally active against strains closely related to the producing strain although there are examples of broader spectrum bacteriocins [9]. LAB-produced bacteriocins are considered ideal for use with food for the following reasons: (1) LAB byproducts are categorized by the FDA as GRAS (generally recognized as safe) [10]; (2) they are odorless and colorless; (3) they do not impact the organoleptic and sensorial characteristics of food; (4) unlike traditional antibiotics, bacteriocins are cleared by the digestive system by proteolytic enzymes [11]. In addition, bacteriocins have the potential to be bioengineered for improved performance [11]. 

There are two major classes of bacteriocins. Class I bacteriocins, such as lactoccocin, function primarily by inhibiting peptidoglycan synthesis. Class II bacteriocins, such as nisin, function by destabilizing the cytoplasmic membrane, via the creation of pores [12]. With the latter, bacteriocin molecules are absorbed on the membrane surface and form transient pores. This leads to the loss of proton motive force, which alters the permeability of the membrane causing leakage of small nutrient molecules into the surrounding environment, a process which kills the cells. In addition, some bacteriocins function as lysin [13], which degrades the bacterial cell wall, typically composed of peptidoglycan, thus leading to cell lysis.

Many studies reported the inhibitory effects of diverse LAB bacteriocins against a wide variety of food pathogens. For example, the lactobacillin bacteriocin XH1 inhibits the proliferation of *Staphylococcus aureus* and *Escherichia coli* [14], and plantaricin P1053 (produced by *L. plantarum* PBS067) demonstrates a broad-spectrum antimicrobial activity against Gram-negative bacteria, such as *E. coli*, and the Gram-positive *S. aureus.* Bacteriocins are powerful and promising antimicrobials. Therefore, bacteriocin-producing LAB are promising strains for use in food safety applications. Nevertheless, despite their promise, the only LAB bacteriocin that has been approved for use in food by the FDA is nisin [15]. However, many other bacteriocins are being developed for use in food (Table 1).

(b) LAB produce different organic acids that have non-specific antimicrobial effects (Table 2). For example, organic acids, such as acetic acid, propionic acid, and lactic acid, are synthesized by some species of *Acetobacter aceti*, *Propionibacterium*, and *Lactobacillus*, respectively. Other typical organic acids produced by LAB are formate and succinate. Organic acids are attractive because they impede the growth of Gram-negative and Gram-positive bacteria, as well as yeast and molds in several food products. Furthermore, organic acids are generally regarded as safe for human use. Organic acids have antimicrobial properties, and this property has been attributed to dissociated molecules that are deprotonated upon entry into cellular membranes. Another cause of the antimicrobial action of LAB is generally organic acids and this could be due to the concerted effect of both dissociated ions and undissociated molecules, that result in cellular injury. LAB strains are very promising for several food applications due to the synthesis of important metabolites, such as organic acids. 

(c) Many other small molecules exhibiting antimicrobial effects, such as diacetyl, hydrogen peroxide (H_2_O_2_), and reuterin, are produced by LAB (Table 3). For example, Gram-positive and Gram-negative bacteria have been controlled by diacetyl. A highly effective antimicrobial agent used synergistically with heat is hydrogen peroxide (H_2_O_2_). The bactericidal action is more pronounce when it is combined with heat. The antimicrobial mode of action is created by deactivating key enzymes, which results in a modification of catalytic activity. This deactivation is a result of the dicarbonyl group of diacetyls reacting with arginine in the enzymes [37,38,39].

Under aerobic conditions, LAB synthesize hydrogen peroxide (H_2_O_2_) in the absence of intracellular catalase, pseudocatalase, or peroxidase. H_2_O_2_ has bacteriostatic activity, and its mode of antimicrobial activity is enhanced in raw milk by the stimulation of the lactoperoxidase-thiocyanate system. In the presence of H_2_O_2_ and lactoperoxidase enzymes in raw milk, a hypothiocyanite anion is generated. This compound has the potential to destroy cellular components in Gram-negative bacteria, such as the membrane proteins, due to oxidation of the SH group. Reuterin is synthesized by some species of *L. reuteri*, which are usually small antimicrobial molecules and possess antimicrobial properties that inhibit several Gram-positive and Gram-negative bacteria. Reuterin inactivates key enzymes, such as ribonucleotide reductase. Reuterin is resistant to an array of lipolytic and proteolytic enzymes in foods and has a broad working pH range [40,41].

**Table 3:** Antimicrobial compounds produced by LAB and examples of food pathogens and food application.

Small Molecules	Example of Prominent LAB Producer	Example of Food Pathogen Application	Application in Food
Hydrogen Peroxide	*Lactobacillus johnsonii* [37]	*Escherichia coli O157:H7* [38] *Salmonella enterica* [38] *Listeria monocytogenes* [38]	Lettuce [38]
Reuterin	*Limosilactobacillus reuteri* [40]	*Campylobacter* spp. [40] *Escherichia coli O157:H7* [41]	Meat [40]
Diacetyl	*Streptococcus diacetyl lactis* [42]	*Escherichia coli O157:H7* [43] *Salmonella typhimurium* [43]	Meat [43]

## 4. Prevention of Foodborne Pathogens and Elimination of Food Spoilage Bacteria

The causative agents of most reported foodborne illnesses include pathogenic bacteria, such as *Campylobacter jejuni*, *S. aureus*, *L. monocytogenes*, *E. coli*, and *Salmonella*. spp. Consumer demand for minimally processed foods has obiligated the food industry to search for new methods of ensuring food safety. It can no longer rely on traditional heat treatment methods to create microbiologically safe foods. Minimally processed foods, such as fresh fruits and vegetables, have been shown to contain pathogenic bacteria. LAB may increase the nutritional value of food and also support intestinal health through the production of antimicrobial agents. There are many mechanisms for preventing foodborne pathogens and eliminating food spoilage bacteria, such as producing antimicrobial substances that can prevent adhesion of pathogens to epithelial and mucosal surfaces. One of the probiotic mechanisms of action is the competition for adhesion sites [44]. 

LAB are used as bioprotective agents in fishery products. Wiernasz et al. (2017) [1] evaluated the antimicrobial activity of LAB that impeded six common food spoilage bacteria in seafood products (*Brochothrixthermosphacta*, *Serratiaproteamaculans Shewanellabaltica*, *Photobacteriumphosphoreum*, *Lactobacillus sakei*, and *Hafnia alvei*) and one pathogenic bacterium (*L. monocytogenes*) in a co-culture inhibitory assay. An assessment of antimicrobial and spoilage activity, conducted in salmon and cod juice, elucidated strain-specific sensory and inhibition profiles [1]. LAB prevent the clinging of the pathogenic bacteria to their host cells by strengthening the barrier effect of the intestinal mucosa. Another effect of LAB is the influence of the microbial flora through the synthesis of antimicrobial compounds. Several studies have reported the antimicrobial effects of LAB on foodborne pathogens (Table 4).

LABs are also suitable for inhibiting fungi (molds) that are responsible for food spoilage and mycotoxin production. LABs are endowed with bacteriocin-like substances and produce organic acids that have characteristic fungistatic and fungicidal properties and inhibit fungi and yeast, such as *Aspergillus versicolor*, *Debaryomyces hansenii*, *Penicillium expansum*, *Fusarium culmorum*, *Aspergillus fumigatus*, *Candida parapsilosis*, *Aspergillus niger*, and *Penicillium chrysogenum* [56].

Several genera of fungi such as *Aspergillus*, *Fusarium*, *Penicillium*, *Alternaria*, and *Claviceps* spp. produce mycotoxins. Mycotoxins, such as aflatoxins, fumonisins, ochratoxin, patulin, tricothecenes, and zearalenone, are produced as secondary metabolites. These metabolites exhibit carcinogenic, teratogenic, immunotoxic, neurotoxic, hepatotoxic, and nephrotoxic effects. Of the approximately 400 compounds identified as mycotoxins, 30 of them are considered as problematic for the human health. Therefore, the control of mycotoxin contamination is crucial, either by preventing their production or by detoxification. Mycotoxin control becomes harder because conventional cooking processes cannot destroy all of them. Consequently, food processing methods are needed to eliminate mycotoxins. LABs (*Lacticaseibacillus casei* and *Limosilactobacillus reuteri*) are known effectively bind aflatoxins (AFs) in aqueous solutions. Therefore, the control of these microbes using LAB is a natural method of food preservation [57].

## 5. Bacteriocin-Antimicrobial Synergy

The exponential rate of increase in foodborne diseases and their impact on public health warrants immediate action, such as the adoption of comprehensive surveillance measures and the application of synergistic antimicrobial concepts. Thus, the synergistic effect of bacteriocins in conjunction with various antimicrobial agents, such as organic acids, phenolic compounds, essential oils, and chelating agents (for example, EDTA), have proven to be highly effective and thus have been recommended by many authors, due to these products’ extensive spectra of antimicrobial action [58,59]. Bacteriocins are considered as great alternatives to the use of chemical preservatives in dairy products, due to the fact that they are safe antimicrobial peptides. Most bacteriocin producers belong to LAB and they do not pose any health risk concerns. Their mechanism of action is based on their ability to target the cell membrane, DNA, or protein metabolism of the microbial strain, whose function is distinct from those used by antibiotics. Besides, bacteriocins can also be used in the dairy industry in order to enhance fermentation, accelerating cheese ripening, and improve its flavor [60]. One recent technology approaching the problem, that has gained attention and widespread use, is referred to as hurdle technology. Hurdle technology developed as a result of the emergence of physical treatments, such as pulsed electric field, modified atmosphere packaging, gamma irradiation, and heat treatments, in combination with bacteriocins to control food spoilage and pathogenic bacteria [60]. Thus, a major factor that should be considered with regard to the adoption of bacteriocins in a hurdle technological approach is the food matrix and its inherent microflora [61,62]. Bacteriocins, alone or combined with other treatments, may enhance the microbiological safety and improve the sensory properties in dairy products. Several studies have reported the effect of the hurdle technology in controlling pathogens [63]. For example, Sivarooban et al. (2008) [64] have shown that nisin blended with a grape seed extract (GSE) or an extract from green tea (GTE) was effective in damaging targeted cells of a strain of *L. monocytogenes* [64]. Both GSE and GTE contained purified phenolic compounds (GSE: 0.02% catechin and 0.02% epicatechin; GTE: 0.02% epicatechin and 0.02% caffeic acid), respectively, and complemented the antimicrobial action of nisin [64]. Another study reported the effect of combining bacteriocins with chelating agents, such as EDTA, whereby a complex of nisin–sodium–diacetate–EDTA and a combination of nisin–potassium sorbate–EDTA were potent in decreasing the population of *L. monocytogenes* on a shrimp sample containing the pathogen [65]. Other authors reported the inhibitory activity of nisin of over 90% of *L. monocytogenes* in minimally processed ready-to-eat foods, such as lettuce [66].

A study by Branen and Davidson (2004) [67] also confirmed the synergistic effect of nisin and EDTA (at very low concentrations) that were highly effective against *L. monocytogenes* [67]. Consequently, organic acids have been reported to be highly effective in combination with bacteriocins in controlling foodborne pathogens. According to Moon et al. (2002) [68] a bacteriocin from *Pediococcus acidilactici* K10 that was mixed with succinic acid, lactic acid, and acetic acid was synergistically effective against target cells of *E. coli* O157:H7 in vivo and in vitro [68]. Moreover, the same bacteriocin from *Pediococcus acidilactici* K10 mixed with 0.25% acetic acid and 0.35% lactic acid decreased the population of *E. coli* O157:H7, inoculated in a ground beef sample [68]. Therefore, it is noteworthy that a combination of organic acids with bacteriocins is a very promising alternative for bio-preservatives [63].

## 6. Competitive Growth Interactions between LAB and Other Microorganisms

Microorganisms are encompassed by several strains and various species, resulting in intense competition for limited nutrients in their niche. Microbes generally compete for environmental nutrients in two ways. The first is through passive competition, namely exploitation, and the second is by severe competition. Species compete directly for resources, with one species demonstrating higher consumption of resources and thus limiting the other. The second type of competition is direct competition (interference competition), whereby individual cells harm each other by positioning themselves in a state to ward off already established competitors or to entirely destroy competitors, as well as through territorial colonization, especially in uninhabited areas. Many microorganisms are shielded from environmental and microbial risks due to biofilm formation. Biofilms serve as protective barrier for microbial cells. Antimicrobial production is a classic example of competition, whereby antimicrobial agents synthesized from distinct strain-specific bacteriocins have a broad-spectrum effects [7,34].

Due to the growing demand from the food industry to meet the consumer needs of long shelf-life foods that are safe to eat and able to maintain their nutritional and organoleptic qualities, research is increasingly focusing on the role of antimicrobial compounds secreted by natural compounds (such as LAB) as a defense mechanism against intestinal pathogens. LAB can inhibit harmful microorganisms through a competitive exclusion mechanism, based on competition for binding sites and nutrients. Lactic acid bacteria are used to produce fermented products, such as yogurt butter, cheese, kefir, sauerkraut, buttermilk, brined vegetables, sourdough, soya curd, koumiss, idly batter, fermented meat products, and beverages [27]. Several authors investigated the potential applications of lactic microbiota in food products such as milk [69], yogurt [70], cheddar cheese [71], gouda cheese [72], semi-hard Vidiago cheese [73], tenerife cheese [5], italian soft cheese [74], greek feta cheese [75], camembert cheese [76], fresh meat [77], dry-fermented sausages [78], fermented vegetable products [79], fermented vegetables, and fruit drinks [80] in order to improve the products’ safety and quality while preventing pathogenic microorganisms.

In addition, among the outstanding benefits and contributions of lactic acid bacteria, is their use as potential alternatives to antibiotics (in the fight against clinical and subclinical infectious diseases) in the agricultural sector in poultry, pigs, ruminants, and aquacultures [78].

For example, Adeyemo et al. [81] determined the antimicrobial substances produced by LAB isolated from ‘pupuru’ (African traditional cassava food) against food borne pathogens. They reported that four species were tested for antagonistic activity and *L. plantarum* showed the highest zone of inhibition with respect to *S. aureus* and *Pseudomonas aeruginosa*, while the antimicrobial activity decreased with time. Additionally, Rahmeh et al. [52] characterized the antimicrobial traits of LAB in raw camel milk. According to their findings, *Enterococcus*, *Lactococcus*, *Pediococcus* genera, and bacteriocins obtained, exhibited inhibitory activity against a broad spectrum of Gram-positive and Gram-negative bacteria, including multi-drug-resistant *Salmonella*. Among them, *Pediococcus pentosaceus* CM16 and *L. brevis* CM22 were selected for their strong bacteriocinogenic antilisterial activity. *Carnobacterium* spp., *Lactobacillus* spp., *Leuconostoc* spp., *Weissella* spp., and *Enterococcus* spp. were the genera isolated from ready-to-eat seafood (cold-smoked salmon, gravlax, and sushi). LAB isolated from sushi demonstrated a significantly higher antimicrobial effect than LAB from cold-smoked salmon and gravlax [82].

As a result of the quantitative evaluation of *E. coli*, LAB dairy starter culture, and *S. aureus*, it became obvious that the LAB starter culture demonstrated the ability to induce an early stationary phase of *S. aureus* and *E. coli* populations at varying temperatures (for example, from 12 to 37 °C) and for the inocula of lactic acid bacteria (estimated at 10^3^ to 10^6^ CFU/mL) [83].

*L. lactis* subsp. *lactis* NCK401, a LAB strain, was employed as a prototype system in competition with *L. monocytogenes* F5069B, a pathogenic bacterium, in an extract from vegetable broth. Results confirmed the minimum inhibitory concentration of lactic acid to be 6.43 mM for *L. monocytogenes* in a medium of cucumber juice with a stable pH at 5.6 and an ionic strength of 0.342. It was, however, observed that the growth limiting factor for the mixed culture, as well as that of *L. monocytogenes* in the pure culture, was due to pH. Consequently, the growth of the competing bacteria is highly impeded by lactic acid. The inhibitory mechanism of lactic acid (an organic acid) has been explained to be due to the protonated form of the acid that easily permeates and crosses cellular membranes, as they carry no charged ions. Therefore, this results in the conglomeration of acid anions within the cell, leading to the acidification of the cytoplasm and ultimately setting off growth inhibition [9].

## 7. Conclusions

Lactic acid bacteria (LAB) are utilized in the production of a number of fermented foods that provide health benefits and can help to extend the shelf-life of food products. LAB also produce natural antimicrobial compounds that improve the safety of food. For example, LAB produce organic acids, such as lactic, acetic, and formic acids, which cause a drop in pH and thereby inhibit the growth of foodborne pathogens. LAB also produce several antimicrobial compounds, such as bacteriocins. Moreover, LAB are live microorganisms that confer probiotic benefits in the human gastrointestinal tract. In the present review, we discussed a wide range of LAB potential applications in different food products. With regard to our findings in this review, future research should be directed toward novel applications of LAB, including the isolation and selection of new strains. Moreover, the selection of LAB strains with probiotic effects, such as those that produce essential fatty acids, amino acids, vitamins, and functional enzymes (such as lactase), is of importance to both the food industry and consumers. Such new strains can then be adapted for various food applications. In addition, there is a need for the isolation of LAB that can produce unique antimicrobial compounds with a wide range of inhibitory effects against pathogenic microorganisms.

## Figures and Tables

**Table 1:** LAB bacteriocins and their food applications.

Bacteriocin	Strain	Food Applications	Reference
Nisin	*Lactococcus lactis*	Milk	[16]
		Lobster	[17]
		Trout	[18]
		Apple Cider	[19]
		Liquid Whey	[20]
Lacticin	*Lactococcus lactis*	Milk	[21]
		Pork sausage	[22]
Reuterin	*Limosilactobacillus reuteri*	Skim milk	[23]
Gassericin	*Lactobacillus gasseri*	Custard Cream	[24]
Lactococcin	*Lactococcus lactis*	Milk	[25]
Enterocin	*Enteroccocus* spp.	Apple juice	[19]
		Ready to eat salad	[26]

**Table 2:** Organic acids and their applications in foods.

Organic Acid	Example of Prominent LAB Producer	Example Food Pathogen Application	Application in Food
Lactic acid	*Lactobacillus delbrueckii* subsp. *bulgaricus* [27]	*Pseudomonas* spp. [28]	Sliced Salmon [28]
Formic acid	*Lactococcus lactis* subsp. *cremoris* [29]	*Esherichia coli* [30] *Listeria* spp. [30] *Salmonella* spp. [30] *Clostridium perfringens* [31]	Poultry [30] Animal Feed [30] Pork [31]
Succinic acid	*Lactococcus lactis* subsp. *lactis* [29]	*Salmonella* spp. [30]	Chicken meat [32]
Malic acid	*Limosilactobacillus reuteri* [33]	*Staphylococcus* [27,33]	Meat products [33]
Propionic acid	*Lactococcus lactis* subsp. *lactis* [29]	*Campylobacter* spp. [34]	Poultry Food [35]
Acetic acid	*Lactobacillusacidophilus* [29]	*Pseudomonas* spp. [28]	Sliced Salmon [28]
Butyric Acid	*Lactobacillusacidophilus* [29]	*Salmonella* spp. [36]	Poultry [36]

**Table 4:** Inhibition of food pathogens by lactic acid bacteria.

Foodborne Pathogen	Lactic Acid Bacteria	Reference
*Staphylococcus aureus*	*Lactococcus* spp., Pediococcus spp., Lactobacillus spp., Weissella spp., and *Enterococcus* spp. *Lactobacillus curvatus*, *Lactiplantibacillus plantarum*, *Lactobacillus sakei*, *Pediococcus acidilactici,* and *Pediococcus pentosaceus* (industrial products) *Levilactobacillus brevis*, *Lactobacillus coryniformis*, *Lactobacillusparacasei*, *Lactobacillus paraplantarum*, *Leuconostoc mesenteroides,* and *Weisella halotolerans* (traditional products) *Enterococcus faecium* QPII, *Lactiplantibacillus plantarum* CC10, *Lactiplantibacillus plantarum* TF711, *Lacticaseibacillus rhamnosus* IMC 501, *Lacticaseibacillus paracasei* IMC 502, *Lactobacillus sakei* KTU05-6, *Lactobacillus helveticus* KLDS 1.8701, *Pediococcus acidilactici* KTU05-7, *Pediococcus pentosaceus* KTU05-8, KTU05-9, and KTU05-10	[45,46,47,48,49,50]
*Listeria innocua*	*Lactobacillus curvatus*, *Lactiplantibacillus plantarum*, *Lactobacillus sakei*, *Levilactobacillus brevis*, *Lactobacillus coryniformis*, *Lacticaseibacillus paracasei*, *Lactobacillus paraplantarum*, *Leuconostoc mesenteroides*, *Pediococcus acidilactici*, and *Pediococcus pentosaceus* (industrial products), *Weisella halotolerans* (traditional products)	[45,47]
*Escherichia coli*	*Lactobacillus curvatus*, *Lactiplantibacillusplantarum*, *Lactobacillus sakei*, *Levilactobacillus brevis*, *Lactobacillus coryniformis*, *Lacticaseibacillus paracasei*, *Lactobacillus paraplantarum*, *Lactobacillus helveticus* KLDS 1.8701, *Limosilactobacillus reuteri*, *Leuconostoc mesenteroides*, *Pediococcus* spp. *Acidilactici*, *Pediococcus pentosaceus* (industrial products), *Weisella halotolerans* (traditional products)	[45,47,50,51]
*Salmonella enteritidis*	*Limosilactobacillus reuteri*	[45,50,51,52]
*Salmonella typhimurium*	*Levilactobacillus brevis* CM22, *Lactobacillus helveticus* KLDS 1.8701, *Pediococcus pentosaceus* CM16	
*Salmonella cholerae*	*Levilactobacillus brevis* CM22, *Lactobacillus helveticus* KLDS 1.8701, *Pediococcus pentosaceus* CM16	[45,50,52]
*Bacillus cereus*	*Lactobacillus sakei* KTU05-6, *Lacticaseibacillus rhamnosus* IMC 501, and *Lacticaseibacillus paracasei* IMC 502, *Lactiplantibacillus plantarum* TF711, *Pediococcus acidilactici* KTU05-7, *Pediococcus pentosaceus* KTU05-8, KTU05-9, and KTU05-10	[49,53]
*Pseudomonas*	*Lactobacillus sakei* KTU05-6, *Lactobacillus curvatus*, *Lactiplantibacillus plantarum*, *Levilactobacillus brevis*, *Lactobacillus coryniformis*, *Lacticaseibacillus paracasei*, *Lactobacillus paraplantarum*, *Leuconostoc mesenteroides*, *Pediococcus acidilactici* KTU05-7, *Pediococcus pentosaceus* KTU05-8, KTU05-9, and KTU05-10, *Weisella halotolerans* (traditional products)	[47]
*Enterococcus faecium* DSM 13590	*Lactobacillus sakei* KTU05-6, *Lacticaseibacillus rhamnosus* IMC 501 and *Lacticaseibacillus paracasei* IMC 502, *Pediococcus acidilactici* KTU05-7, *Pediococcus pentosaceus* KTU05-8, KTU05-9, and KTU05-10	[46,47]
*Listeria monocytogenes*	*Enterococcus faecium* QPII, *Lactobacillus sakei* KTU05-6, *Lacticaseibacillus rhamnosus* IMC 501 and *Lacticaseibacillus paracasei* IMC 502, *Lactobacillus curvatus*, *Levilactobacillus brevis*, *Lactobacillus coryniformis*, *Lacticaseibacillus paracasei*, *Lactobacillus paraplantarum*, *Lactiplantibacillus plantarum* CC10, *Lactobacillus helveticus* KLDS 1.8701, *Limosilactobacillus reuteri*, *Leuconostoc mesenteroides*, *Pediococcus acidilactici* KTU05-7, *Pediococcus pentosaceus* KTU05-8, KTU05-9, and KTU05-10, *Weisella halotolerans* (traditional products)	[46,48,50,54]
*Clostridiumsporogenes*	*Lactiplantibacillus plantarum* TF711	[49]
*Shigella sonnei*	*Lactiplantibacillus plantarum* TF711	[49]
*Klebsiella pneumoniae*	*Lactiplantibacillus plantarum* TF711	[49]
*Acinetobacter baumannii*	*Lactobacillus* spp.	[55]

## Data Availability

No new data were created or analyzed in this study.
